# Composition, anti-LDL oxidation, and non-enzymatic glycosylation inhibitory activities of the flavonoids from *Mesembryanthemum crystallinum*

**DOI:** 10.3389/fnut.2022.963858

**Published:** 2022-09-14

**Authors:** Meiling Sun, Ying Wei, Xiaoguang Feng, Junfeng Fan, Xiangning Chen

**Affiliations:** ^1^Department of Food Science and Engineering, College of Food Science and Engineering, Beijing University of Agriculture, Beijing, China; ^2^Beijing Unong High-Quality Farm Products Planning Limited Company, Beijing, China; ^3^Beijing Key Laboratory of Forest Food Processing and Safety, Department of Food Science and Engineering, College of Biological Sciences and Technology, Beijing Forestry University, Beijing, China

**Keywords:** flavonoid compounds, UPLC-MS/MS, low-density lipoprotein, oxidation, non-enzymatic glycosylation

## Abstract

This study identified the constituents of purified flavonoid (PEF) isolated from *Mesembryanthemum crystallinum* and examined their inhibitory effects on low-density lipoprotein (LDL) oxidation and non-enzymatic glycosylation. More than 30 kinds of flavonoid compounds were identified in *M. crystallinum*, including tangeretin, nobiletin, farrerol, protocatechuic aldehyde, diosmin, and rutin. Moreover, tangeretin corresponds to approximately 51% of the total identified flavonoids. PEF had a low IC_50_ value for 1,1-diphenyl-2-picrylhydrazyl radicals (DPPH·), hydroxyl radical (·OH), and superoxide anion free radical (O${}_{2}^{-}\cdot$) scavenging. They were found to effectively delay and inhibit the production of conjugated diene (CD) and thiobarbituric acid reactive substance (TBARS) during LDL oxidation. Meanwhile, scanning electron microscopy (SEM) of the LDL oxidation incubation system with PEF showed a smooth and dense surface, with no obvious cavitation phenomenon. Furthermore, PEF effectively inhibited the production of LDL glycosylation products and showed a strong inhibitory effect in the latter stage. The electrophoresis of advanced glycosylation end products (AGEs) further confirmed that PEF can effectively prevent the cross-linking between glucose and proteins, protecting LDL from glycosylation-induced damage.

## Introduction

*M. crystallinum* is a green vegetable from Japan introduced to China in recent years. This vegetable contains a large quantity of nutrients, such as basic minerals (sodium, calcium, magnesium, and iron), amino acids, malic acid, inositol, and anti-acidification substances. It thus helps in quenching thirst, detoxification, and digestion (1). The alkaline plant salt contained in *M. crystallinum* has significant clinical therapeutic effect on some common diseases, such as high blood lipid and blood pressure, diabetes mellitus, obesity, immune dysfunction and tumor, which are sensitive to sodium (2). Li et al. (3) studied the chemical components in *M. crystallinum* and isolated 19 compounds including stigmasterol, ryegrass lactone, cis-*p*-hydroxycinnamic acid, niacin, and ferulic acid. As a nutritious green vegetable, *M. crystallinum* has a high flavonoid content (4). Flavonoids are secondary plant metabolites and have antioxidant and anti-tumor properties (5). However, the antioxidative potential of glycoside-free flavonoids [e.g., purified flavonoid compounds (PEF) from *M.crystallinum*] against low-density lipoprotein (LDL) has not been reported. Furthermore, anthocyanins can directly transmit antioxidant effects through their antioxidant properties, and indirectly combat oxidative stress and inflammatory signals in cells within atherosclerosis (AS) plaques by inducing nuclear factor erythroid 2-related factor 2 (Nrf2) activation and antioxidant gene expression, thus effectively preventing AS (6).

Cardiovascular disease (CVD), which is mainly caused by AS, is a major threat to human health. CVD has a high incidence and high mortality rate and affects millions of people worldwide (7). Previous studies have reported that LDL is most susceptible to oxidation and non-enzymatic glycosylation. Thus, elevated LDL levels are a significant risk factor for AS. This suggests that AS can be prevented by inhibiting LDL oxidation and glycosylation (8). Conventionally, statins, ezetimibe, and other lipid-lowering and antidiabetic drugs such as metformin have been used to reduce LDL levels. However, these drugs cause several adverse effects, including liver dysfunction. Therefore, natural ingredients extracted and purified from plants, such as polysaccharides, polyphenols, and terpenoids, have been extensively studied due to their protective effects against AS and their safety. Therefore, natural active ingredients extracted and purified from plants are extensively being researched for their protective effects against AS as a result to their safety. Flavonoids, a type of polyphenols, have strong antioxidant activity and have received a lot of attention from researchers. Therefore, the discovery of flavonoid compounds that inhibits LDL oxidation and glycosylation is particularly important for the prevention of AS and CVD. Recent studies have proven that plant-derived flavonoids such as soybean isoflavones can hinder LDL oxidation and reduce the degree of oxidation-induced modifications, thus mitigating the peroxidative damage caused by free radicals (9, 10). However, glycosides, such as glucoside, acetyl glucoside, and malonyl glucoside, correspond for about 97–98% of the total isoflavones identified in soybean (11, 12). In addition, LDL oxidation is a complex multi-stage process (13). Previous research has elucidated the physiological function of soybean isoflavones, but studies on their inhibitory effects against LDL oxidation have been limited. These studies have only examined malondialdehyde (MDA), which is insufficient to explain the entire problem. Moreover, whether flavonoids can inhibit LDL glycosylation [e.g., inhibiting the production of Amadori products, dicarbonyl compounds, and advanced glycosylation end products (AGEs) at all stages] and thus prevent AS has not been investigated to date. Further, in these studies, the antioxidative effects of the glycosides present cannot be ruled out.

Therefore, we hypothesized that the PEF, which don't contain glycosides could inhibit the oxidation and glycosylation of LDL, and studied their inhibitory effects at different stages. Because no such study has been done before, this study could not only lay the theoretical foundation for insights into the function of flavonoid compounds but also provide new ideas and directions for delaying LDL oxidation and glycosylation, which has important practical significance for AS prevention. This research mainly focused on CD and TBARS generating inhibition rate during LDL oxidation, and Amadori products, dicarbonyl compounds, and AGEs generating inhibition rate at all stages during LDL glycosylation, which applied to UV spectroscopy, sodium dodecyl sulfate-polyacrylamide gel electrophoresis (SDS-PAGE) and scanning electron microscopy (SEM).

## Materials and methods

### Materials

Fresh *M. crystallinum* was purchased from the Beijing Agricultural College market. Fresh pig's blood was commercially acquired from Beijing Langzhong Slaughterhouse, which is a regular commercially available product in China, in compliance with laws and regulations. The M5 5 × SDS-PAGE Protein sampling buffer and M5 Prestained Protein Ladder (10–180 kDa) were purchased from the Beijing Polymei Biotechnology Co., LTD. The tangeretin standard (≥95%), Rutin standard (HPLC grade), and vitamin C standard (HPLC grade) were all purchased from Beijing Solaibao Technology Co., LTD. The total protein quantitative test box were purchased from Nanjing Jiancheng Bioengineering Institute. All other reagents were of analytical grade.

### Extraction of flavonoids

The *M. Crystallinum* was dried at 60°C for 48 h by vacuum drying oven (DZF-1, China), and then smashed and sieved over a 60-mesh to obtain a powder. Flavonoids were extracted using a previously described method (14, 15). Briefly, the powder was mixed with 70% ethanol (1:25, w/v, g/mL) for 30 min and ultrasonically extracted at 250 W and 60°C for 120 min by ultrasonic cleaners (KQ-500DE, China). It was then centrifuged at 10,000 rpm for 15 min by high speed freezing centrifuge (Sorvall ST16R, USA). The supernatant was collected and evaporated under reduced pressure (RE-52AA, China). Then, it was freeze-dried at −60°C for 24 h to obtain the crude flavonoids by Ultra-low Freeze Dryer (LyoMotion LAB, Germany), which were named CEF.

### Purification of flavonoids

Purification was performed by macroporous resin method (16), according to the conditions referred to by Sun et al. (17). Finally, the eluent was collected and evaporated under reduced. Accordingly, purified flavonoids, which were named PEF.

### Mass concentration determination of flavonoids

The total flavonoid content in the CEF and PEF was determined according to the method previously described by Nayeem et al. (18). The NaNO_2_-Al(NO_3_)_3_ colorimetric method was used, with rutin as the standard. The linear regression equation of the standard curve was y = 0.102x + 0.0002; R^2^ = 0.9998. The sample powder was redissolved in 60% ethanol, and the absorbance was determined using the standard curve method. Accordingly, the total flavonoid content was calculated.

### Composition determination of flavonoids

The composition and relative percentage of the flavonoids in PEF were detected using UPLC-MS/MS (XCIEXAD, USA) (17). First, 200 mg PEF powder was dissolved in 100 mL ethanol (volume fraction of 80%). Then, ultrasonic treatment was performed for 30 min, with eddy shaking. The solution was then centrifuged at 12,000 rpm at 4°C for 20 min. Finally, the supernatant was collected and analyzed. Data were collected in the positive and negative modes. The correlation analysis for the *m/z* value, retention time, and peak area of each mass spectrum peak was conducted using the MRM mode. Based on the identification of the precursor ions and the product ion, the specificity of the precursor ion was detected. The corresponding specific temperament ion was selected, and the other product ion disruptions were removed. Based on information from the literature and the flavonoids standard database, the specific composition of PEF was determined (19). To calculate the relative percentage content, the internal standard method was used, which is a common quantitative analysis method in chromatographic analysis. A known amount of internal standard (2-chloro-L-phenylalanine) was used for chromatographic analysis, and the internal standard was separated into peaks on the chromatogram. Based on the peak area of the known concentration of the internal standard and the peak area of the sample, the relative percentage content of each compound was calculated (20). The column used was a UPLC BEH C18 column (1.7 μm, 2.1 × 150 mm). The mobile phase was 0.1% formic acid aqueous solution (phase A) + acetonitrile (phase B), the column temperature was 40°C, the temperature of the automatic sampler was 8°C, and the sample volume is 2 μL. The mass spectrometry parameters used were AB Sciex QTrap 6500+ and the mass spectra were used in multiple reaction monitoring (MRM) mode during data acquisition. The remaining conditions were IonSpray Voltage: +5,000/−4,500 V, Curtain Gas: 35 psi, Temperature: 500°C, Ion Source Gas 1: 55 psi, Ion Source Gas 2: 60 psi. The mobile phase conditions of liquid chromatography as shown in Table 1.

**Table 1:** Mobile phase conditions of liquid chromatography.

**Time (min)**	**Flow velocity (μL/min)**	**A phase (%)**	**B phase (%)**
0	300	90	10
0.5	300	90	10
15	300	40	60
16.01	300	2	98
18.00	300	2	98
18.01	300	90	10
20.00	300	90	10

### Determination of DPPH·, ·OH and O${}_{2}^{\text{–}}\cdot$ scavenging capacity

The DPPH·, ·OH, and O${}_{2}^{-}\cdot$ scavenging capacities of the samples were examined according to previously described methods (21–23). In addition to CEF and PEF, the components with the highest relative percentage of PEF were set as the other group, which was named tangeretin (GNT). Vitamin C (Vc) was used as a positive control, and the following concentrations were prepared in 60% ethanol: 0.01, 0.02, 0.04, 0.06, 0.08, and 0.10 mg/mL. The scavenging capacity was determined based on the clearance rate (%), meanwhile, the IC_50_ of each group was also calculated.

A 0.1 mL of sample solution with different concentrations was measured in the test tube, and 3.9 mL of DPPH-ethanol solution with 0.1 mmol/L was added, respectively. The mixture was mixed and left for 30 min at 25°C in the dark. The mixture was centrifuged at 4,000 rpm for 5 min, the absorbance A_1_ of the supernatant was measured at 510 nm. The DPPH· clearance rate was calculated based on the following equations:


(1) $$\text{DPPH·clearance rate }(\%)=(\text{1}-\frac{{\text{A}}_{1}{-\text{ A}}_{\text{2}}}{{\text{A}}_{0}})\times \text{100}$$

where, the equal-volume anhydrous ethanol instead of the DPPH-ethanol solution was measured A_2_; A_0_ was measured by equal volume deionized water instead of the sample liquid. The standard Vc with equal concentration served as the positive control group.

A 1 mL of sample solution with different concentrations was measured in the test tube, and 8 mmol/L FeSO_4_ and 1 mL H_2_O_2_ were added, respectively. Then left it to stand at 25°C for 10 min. Finally, 1 mL of 8 mmol/L salicylic acid-ethanol solutions was added and after mixing, the supernatant was centrifuged at 4,000 rpm for 5 min at a constant temperature of the water bath at 37°C for 1 h. The absorption value of the supernatant was measured at 510 nm and recorded as A_2_. The ·OH clearance rate was calculated based on the following equations:


(2) $$\text{·OH clearance rate }(\%)=(1-\frac{{\text{A}}_{2}{-\text{A}}_{\text{1}}}{{\text{A}}_{0}})\times \text{100}$$

where, A_1_ was measured by replacing H_2_O_2_ with deionized water in equal volumes. A_0_ was measured by equal volumes of deionized water instead of sample liquid. The standard Vc of equal concentration was the positive control group.

A 4.5 mL of 50 mmol/L Tris-HCl buffer solution (pH 8.2) was measured, at a constant temperature water bath at 25°C for 30 min. Then, 0.4 mL of 25 mmol/L gallic acid solution and 1 mL of sample solution different concentration, as shown above, were added to each solution, and the mixture was mixed in a constant temperature water bath at 25°C for 5 min. Then 1 mL of 8 mmol/L hydrochloric acid solution was added and centrifuged at 4,000 rpm for 5 min, and the absorption value of the supernatant was measured at 320 nm and recorded as A1. The O${}_{2}^{-}\cdot$ clearance rate was calculated based on the following equations:


(3) $${\text{O}}_{2}^{-}\cdot \text{ clearance rate }(\%)=(1-\frac{{\text{A}}_{1}}{{\text{A}}_{0}})\times \text{100}$$

where, A_0_ was measured by using equal volumes of deionized water instead of the sample liquid. The standard Vc of equal concentration was the positive control group.

### Preparation of LDL

Plasma LDL was precipitated using the sodium citrate–heparin precipitation method (24). The total protein concentration was determined using the total protein quantitative test box. The solution was diluted to the required concentration using PBS and then stored at 4°C for 2 weeks.

LDL was further identified using SDS-PAGE; 12 and 4% acrylamide was used for the separating and stacking gels, respectively (25). Here, 5 μL prestained protein ladder (10–180 kDa) and 20 μL pretreated protein sample were added to each well. The prepared LDL solution was diluted 1, 5, 10, 20, and 30 times and mixed with 5× SDS-PAGE protein loading buffer in a 4:1 ratio, boiled for 5 min, and centrifuged at 10,000 rpm for 3 min. The sample was electrophoresed at 80 V for 40 min and then at 200 V until the blue dye reached the bottom of the separation glue. Then, the gel sheet was stained for 1 h using the Coomassie brilliant blue R-250 dye solution and decolorized with methanol-glacial acetic acid on a horizontal decolorization shaking table. Images were collected using a gel image analyzer, and protein bands were observed (26).

### The LDL oxidation incubation system

Based on the detected components and contents of PEF, an oxidation incubation system consisting of n-LDL, ox-LDL, butylated hydroxytoluene (BHT), CEF, PEF, and GNT was established (Table 2).

**Table 2:** Oxidation incubation system of low-density lipoprotein.

**Group (37°C)**	**Oxidation reaction substrate**	**Samples**	**Oxidant**
n-LDL	LDL	Methanol	ddH_2_O
ox-LDL	LDL	Methanol	CuSO_4_·5H_2_O
Positive control (BHT)	LDL	BHT	CuSO_4_·5H_2_O
CEF	LDL	CEF	CuSO_4_·5H_2_O
PEF	LDL	PEF	CuSO_4_·5H_2_O
GNT	LDL	GNT	CuSO_4_·5H_2_O

#### Inhibition of CD production

This assay was performed according to a previously reported method (27). Briefly, the BHT, CEF, PEF, and GNT were prepared as 0.02, 0.06, and 0.10 mg/mL sample solutions in 60% ethanol. The concentration of the LDL solution was adjusted to 600 μg/mL, and the concentration of CuSO_4_·5H_2_O was 50 μmol/L. Based on the above oxidation incubation system and the ratio of 98:1:1, the sample solution was removed every 1 h and centrifuged for 5 min at 10,000 rpm. Then, the absorbance value of the supernatant was determined at 234 nm and recorded as OD_234_. The 24-h CD production and degradation kinetics curves of each group at 0.10 mg/mL were plotted. The maximum value was used to plot the histogram of CD production inhibition by the flavonoids at different concentrations.

#### Inhibition of TBARS production

A slightly modified version of a previously reported protocol was used (28). Briefly, the sample solution was prepared as described in Section Inhibition of CD production. The concentration of the LDL solution was adjusted to 3.2 mg/mL, and the concentration of CuSO_4_·5H_2_O was 300 μmol/L. First, 1 mL sample solution was removed from the incubation system at various time points, i.e., 6, 12, 18, 24, 36, 48, 60, and 72 h. Then, 50 μL 1% EDTA-2Na was added to stop the oxidation reaction. Following this, 1 mL of 15% trichloroacetic acid solution was added to precipitate the protein. The color was obtained by adding 1 mL 0.67% total bile acid (TBA) solution. After boiling in a water bath for 35 min, the mixture was cooled to 25°C and centrifuged at 10,000 rpm for 5 min. The absorbance of the supernatant was measured at 532 nm and recorded as OD_532_. The 72-h TBARS production and degradation kinetics curves of each group at 0.10 mg/mL were plotted. The maximum value was used to plot the histogram of TBARS production inhibition by the flavonoids at different concentrations.

#### Observation of LDL microstructure before and after oxidation

BHT, CEF, PEF, and GNT sample solutions of 0.10 mg/mL were prepared in 60% ethanol. They were incubated with the LDL mixture for 48 h using the protocol described in Section Inhibition of CD production. After incubation, all the sample solutions were added to a regenerated cellulose dialysis bag (retention weight = 50 kDa). Then, different LDL powders were obtained through freeze-drying at −60°C for 24 h. Finally, a small amount of powder from each group was spray-coated with gold, and its microstructure was observed using SEM.

### The LDL non-enzymatic glycosylation incubation system

A slightly modified version of a previously reported protocol was used (29, 30). The AG, CEF, PEF, and GNT samples were prepared using 60% ethanol. The gradient of solution concentrations included 0.02, 0.06, and 0.10 mg/mL. The concentration of the LDL solution was adjusted to 1.5 mg/mL, and the final concentration of the glucose solution was 500 mM. A non-enzymatic glycosylation incubation system was established as shown in Table 3.

**Table 3:** Non-enzymatic glycation incubation system of low density lipoprotein.

**Group (50°C)**	**Glycosylation reaction substrate (LDL)/mL**	**Samples/mL**	**Glucose /mL**	**PBS/mL**	**ddH_2_O/mL**
gly-LDL	4	0	4	2	2
Control A (n-gly-LDL)	4	0	0	2	6
Control B (n-LDL)	0	2	0	4	6
Positive control (AG)	4	2	4	2	0
CEF	4	2	4	2	0
PEF	4	2	4	2	0
GNT	4	2	4	2	0

#### Inhibition rate of early Amadori products formation

A slightly modified version of a previously reported protocol was used (29). A 750 μL sample solution was dissolved in 2.5 mL of 0.3 mM NBT (prepared in a 100 mM sodium bicarbonate-sodium carbonate buffer system; pH 10.35) and incubated in the non-enzymatic glycosylation incubation system. After 7 days, the reaction was terminated by adding 400 μL of 10% glacial acetic acid in a water bath at 37°C for 20 min. The absorbance was measured at 530 nm. Subsequently, the inhibition rates of AG, CEF, PEF, and GNT against early-stage non-enzymatic LDL glycosylation were calculated using the following equation:


(4) $$\text{Inhibition rate }(\%)=\left(1-\frac{{\text{A }-\text{ A}}_{\text{2}}{-\text{ A}}_{\text{3}}}{{\text{A}}_{1}{-\text{ A}}_{\text{2}}}\right)\times \text{100}$$

Where, A is the absorbance value of the AG, CEF, PEF, and GNT experimental groups; A_1_ is the absorbance value of the glycosylation group; A_2_ is the absorbance value of control group A; A_3_ is the absorbance value of control group B.

#### Inhibition rate of the metaphase dicarbonyl compound formation

The above non-enzymatic glycosylation incubation system was incubated with the respective solutions for 14 d. About 200 μL of 500 mM girarte reagent T was first added to 400 μL of a sample solution, and then, 3.4 mL of 500 mM sodium formate buffer (pH 2.9) was added. After mixing, the mixture was placed in a water bath at 37°C for 1 h and centrifuged at 10,000 rpm for 5 min. The absorbance of the supernatant was measured at 294 nm. The inhibition rate for dicarbonyl compounds was calculated using the formula mentioned above.

#### Generation of the late AGEs

A slightly modified version of a previously reported method was used (30). AG, CEF, PEF, and GNT were prepared as 0.10 mg/mL sample solutions in 60% ethanol. The other incubation methods used were the same as those described in Section The LDL non-enzymatic glycosylation incubation system. The specific test conditions and methods used were similar to those described in Section Preparation of LDL.

### Statistical analysis

All experiments were performed in triplicate, and data were presented as the mean value ± standard deviation. SPSS Statistics 21 (Version X, IBM, Armonk, NY, USA) and Origin 8.5 (OriginLab, USA) software were used to analyze and plot the experimental data. The IC_50_ value represented the semi-inhibitory concentration of enzyme inhibitors. It was calculated and analyzed using GraphPad Prism 8 (Graph Pad Software, San Diego, CA, USA). Values were considered significant at *p* < 0.05.

## Results and discussion

### Analysis of the composition of PEF

**Figure 1:**
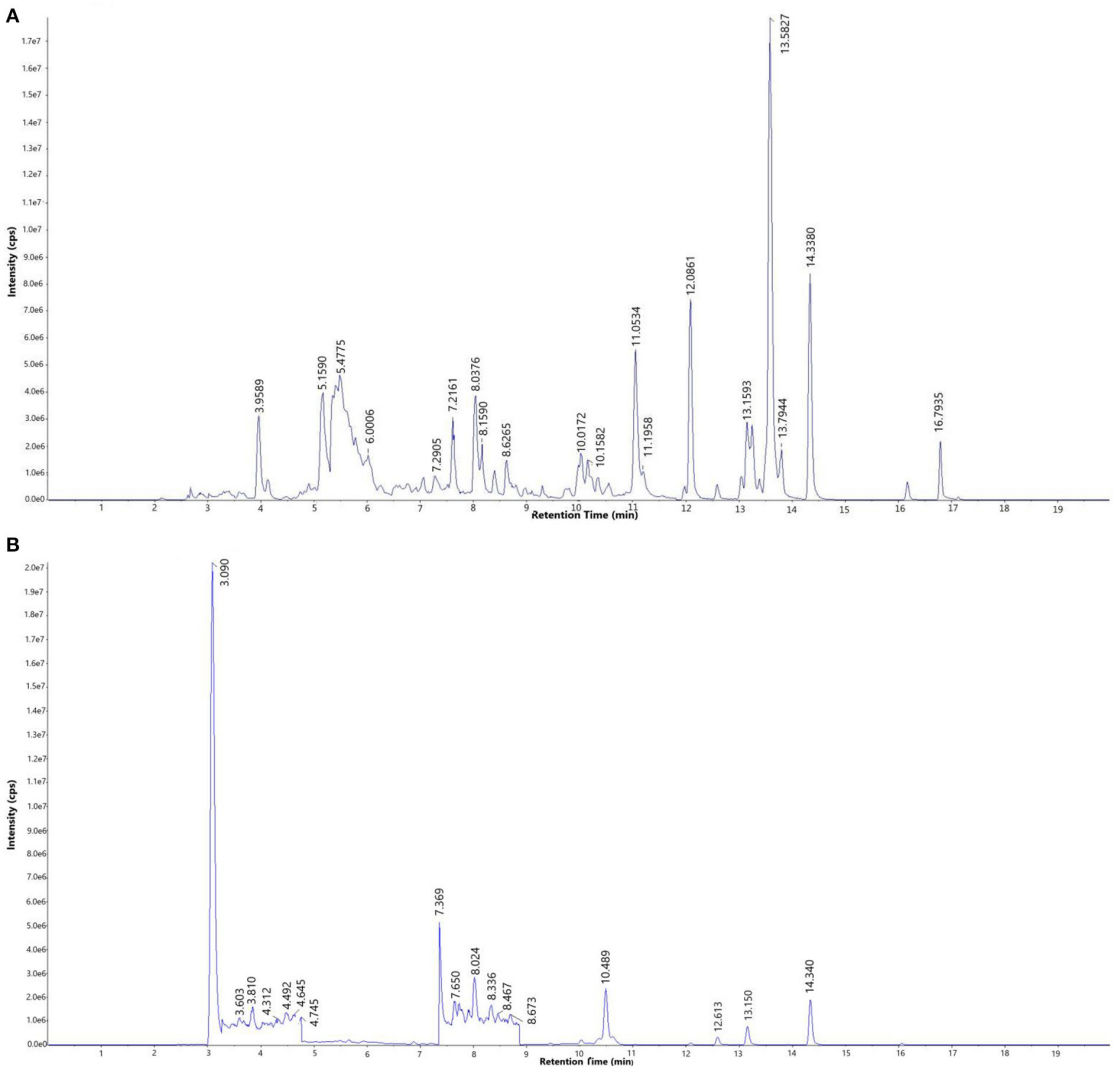
Total ion current chromatogram (TIC) of **(A)** mixed standard and **(B)** purified flavonoids from *M. crystallinum* (PEF).

**Figure 2:**
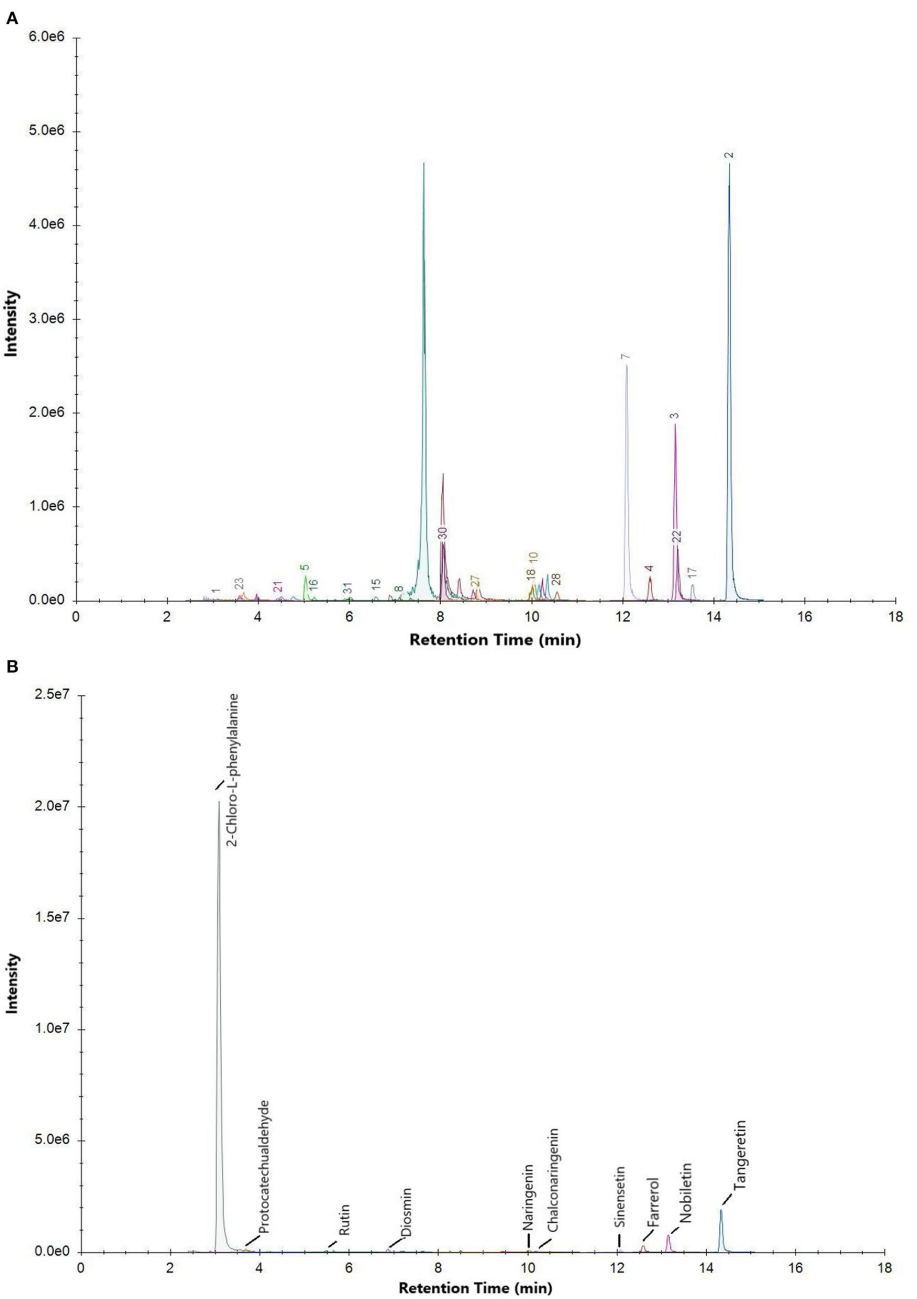
Extracted ion chromatogram (EIC) of **(A)** mixed standard and **(B)** PEF.

The total ion current chromatogram (TIC) of the mixed standard and PEF are shown in Figure 1, and extracted ion chromatogram (EIC) are shown in Figure 2. The MRM ion pair, retention time, and peak area analyzed in the MRM mode are shown in Table 4. The mixed standard was the mixture of authentic standard compounds, and could thus help in the identification of the flavonoids in the sample. However, the ion ranges represented by the TIC and EIC are different. The TIC is the chromatogram of all ions within the set range, and the ordinate represents the addition of all ion intensities (31). A peak does not represent an individual substance. Hence, the detected substance cannot be identified in Figure 1B. In EIC, a specific *m/z* or several *m/z* values are selected on the basis of TIC data, and a chromatogram of its signal intensity is drawn as a function of retention time. Given the high sensitivity of QTRAP mass spectrometry MRM acquisition mode, this mode is usually used for the absolute quantification of substances. Flavonoid broad target is a special detection method for flavonoids. In order to avoid interference from other background substances and better detect the target flavonoids, the high-sensitivity MRM acquisition mode was used to collect flavonoids in a targeted manner. Moreover, high-sensitivity detection was conducted by using the corresponding standards to assist with qualitative detection, thereby improving the reliability of results. MRM technology is widely used due to advantages such as strong specificity, high sensitivity, high accuracy, and good reproducibility (32). During the experiment, we optimized the collision energy (CE) and the fragmentor voltage (FV) of the MRM transitions.

**Table 4:** Components and relative contents of total flavonoids purified from *M. crystallinum*.

**Serial number**	**English name**	**Chemical formula**	**Categories**	**MRM ion pair**			**Retention time /min**	**Peak** **area**	**Relative percentage/%**
				**Precursor ion**	**Ion modes**	**Product ion**			
1	Tangeretin	C_20_H_20_O_7_	Flavonoids	372.8	+	342.9	14.34	8,183,934	50.85 ± 0.09^a^
2	Nobiletin	C_21_H_22_O_8_	Flavonoids	403.4	+	372.8	13.15	3,369,730	20.95 ± 0.12^b^
3	Farrerol	C_17_H_16_O_5_	Flavonol	301.3	+	180.9	12.61	1,413,145	8.78 ± 0.07^c^
4	Daidzin	C_21_H_20_O_9_	Isoflavones	416.9	+	255.0	5.02	65,861	0.42 ± 0.04^i^
5	Protocatechualdehyde	C_7_H_6_O_3_	Flavonol	138.9	+	93.1	3.69	637,589	3.96 ± 0.11^d^
6	sinensetin	C_20_H_20_O_7_	Flavonoids	373.0	+	342.9	12.09	360,637	2.24 ± 0.06^ef^
7	Hesperidin	C_16_H_14_O_6_	Dihydrogen Flavonoids	609.0	–	300.8	7.06	54,493	0.35 ± 0.02^i^
8	Chalconaringenin	C_27_H_34_O_14_	Dihydrochalcone	272.9	+	152.9	10.02	409,064	2.52 ± 0.05^e^
9	Naringenin	C_15_H_12_O_5_	Dihydrogen Flavonoids	273.3	+	152.8	10.04	346,185	2.15 ± 0.07^f^
10	Diosmin	C_28_H_32_O_15_	Dihydrogen Flavonoids	609.1	+	463.0	6.88	462,651	2.88 ± 0.08^e^
11	Rutin	C_27_H_30_O_16_	Flavonol	611.0	+	302.9	5.66	177,213	1.72 ± 0.05^g^
12	Tectorigenin	C_22_H_22_O_11_	Isoflavones	300.9	+	285.5	10.16	146,889	0.913 ± 0.057^h^
13	Quercetin	C_15_H_10_O_7_	Flavonol	300.9	–	150.8	8.80	69,501	0.432 ± 0.013^i^
14	Narirutin	C_27_H_32_O_14_	Flavonol	579.0	–	270.9	6.57	11,438	0.071 ± 0.011^m^
15	Glycitin	C_22_H_22_O_10_	Flavonol	447.4	+	284.9	5.22	6,704	0.042 ± 0.007^n^
16	Pinocembrin	C_15_H_12_O_4_	Dihydrogen Flavonoids	257.3	+	152.8	13.52	32,106	0.200 ± 0.010^k^
17	Apigenin	C_15_H_10_O_5_	Flavonoids	270.9	+	152.9	9.99	32,082	0.199 ± 0.025^k^
18	Eriodictyol	C_15_H_12_O_6_	Dihydrogen Flavonoids	288.9	+	152.9	8.70	37,958	0.235 ± 0.013^j^
19	Isoquercitrin	C_21_H_20_O_12_	Flavonol	465.4	+	302.8	5.97	37,996	0.236 ± 0.021^j^
20	L-Epicatechin	C_15_H_14_O_6_	Flavanol	291.3	+	138.9	4.39	23,331	0.144 ± 0.016^l^
21	Chrysin	C_15_H_10_O_4_	Dihydrogen Flavonoids	255.2	+	152.8	13.22	19,399	0.121 ± 0.007^l^
22	Cianidanol	C_15_H_14_O_6_	Flavanol	290.9	+	138.8	3.58	6,870	0.043 ± 0.008^n^
23	Morin	C_15_H_10_O_7_·2H_2_O	Flavonol	300.8	+	272.8	8.02	10,708	0.067 ± 0.009^mn^
24	(-)-Epicatechin gallate	C_22_H_18_O_10_	Flavanol	440.9	–	168.9	6.00	15,776	0.097 ± 0.003^lm^
25	Kaempferol	C_15_H_10_O_6_	Flavonol	286.9	+	152.9	10.22	16,020	0.100 ± 0.010^lm^
26	Luteolin	C_15_H_10_O6	Flavonol	286.9	+	152.8	8.77	17,310	0.108 ± 0.007^lm^
27	Hesperetin	C_16_H_14_O_6_	Dihydrogen Flavonoids	303.3	+	176.9	10.56	8,501	0.053 ± 0.003^mn^
28	(-)-Epigallocatechin gallate	C_22_H_18_O_11_	Flavanol	457.0	–	168.8	4.46	4,070	0.025 ± 0.005^n^
29	Baicalin	C_21_H_18_O_11_	Flavonoids	447.0	+	270.9	8.05	11,906	0.074 ± 0.009^m^
30	Kaempferitrin	C_27_H_30_O_14_	Flavonol	577.5	–	284.9	5.97	7,027	0.044 ± 0.004^n^

Different superscript alphabets in the table represent significant differences in the relative percentage content of each component (*p* < 0.05).

To determine the relative percentage content of each component, the internal standard method was used. As illustrated in Table 4, a total of 30 flavonoids were detected in PEF. The relative content of tangeretin was the highest, i.e., 50.85%, followed by that of nobiletin, farrerol, protocatechualdehyde, diosmin, chalconaringenin, sinensetin, naringenin, and rutin. In addition, the relative percentages of the other 20 flavonoids were all below 1%, and the EIC peak was not visible. Hence, we only labeled 9 kinds of flavonoids in Figure 2B. Nevertheless, tangeretin and nobiletin together accounted for more than 20% of the content, and the chromatographic peaks were prominent. The relative percentage of (-)-Epigallocatechin gallate was the lowest at 0.03%. Based on the oxidation degree of the three carbon bond (C3) structure and B ring connection sites, the 30 kinds of flavonoid compounds could be divided into six categories (33): flavonoids, dihydrogen flavonoids, flavanol, flavonol, isoflavones, and dihydrochalcone. Of these flavonoid compounds, flavonoids made up a majority of the content—i.e., 74.31%. These results indicated that *M. crystallinum* had a rich variety and high content of flavonoids, and the subsequent biological activity tests would thus have certain research significance. Structurally, most of the detected flavonoids did not contain glycosides. Hence, the results were expected to lay a theoretical foundation for subsequent research on the inhibitory effects of glycoside-free flavonoids on LDL oxidation and glycosylation.

### DPPH· scavenging capacity analysis

**Figure 3:**
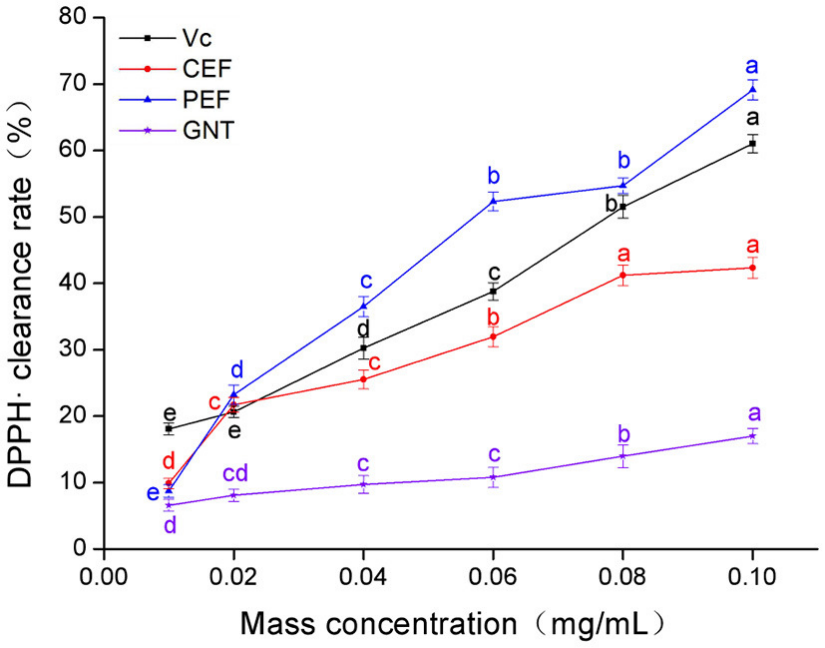
DPPH· clearance rate of CEF, PEF, and GNT at different concentrations. Different letters indicate significant differences in DPPH·scavenging ability of the same substance at different concentrations (*p* < 0.05).

The clearance rate of DPPH· in the experimental groups was dose-dependent (Figure 3). The Vc clearance rate was highest when the sample concentration was 0.01 mg/mL. However, when the concentration range was 0.02–0.10 mg/mL, the clearance rates in each group were in the descending order: PEF, Vc, CEF, and GNT; the IC_50_ values were 0.056, 0.071, 0.189, and 0.428 mg/mL, respectively. The clearance rates of different concentrations of GNT against DPPH· were comparable to the other three groups (*p* > 0.05). GNT was the flavonoid with the highest relative percentage content in PEF, but its clearance rate was significantly lower than that of PEF at the same concentration. Further, the IC_50_ was 7.64 times that of PEF. Extracted from pure natural vegetables, PEF contains a rich variety and content of flavonoids. The scavenging effect of PEF on DPPH· was synergistic, and the antioxidant capacity was higher than that of a single flavone standard. The antioxidant capacity of flavonoids from *M. crystallinum* has so far not been studied. These results indicated that the rate of DPPH· clearance was significantly improved after the purification of PEF, even at the same concentration. The clearance rate achieved by PEF was even higher than that of Vc. When the concentration was 0.10 mg/mL, the clearance rate of PEF was 1.63 times that of CEF and 1.13 times that of Vc.

### ·OH scavenging capacity analysis

**Figure 4:**
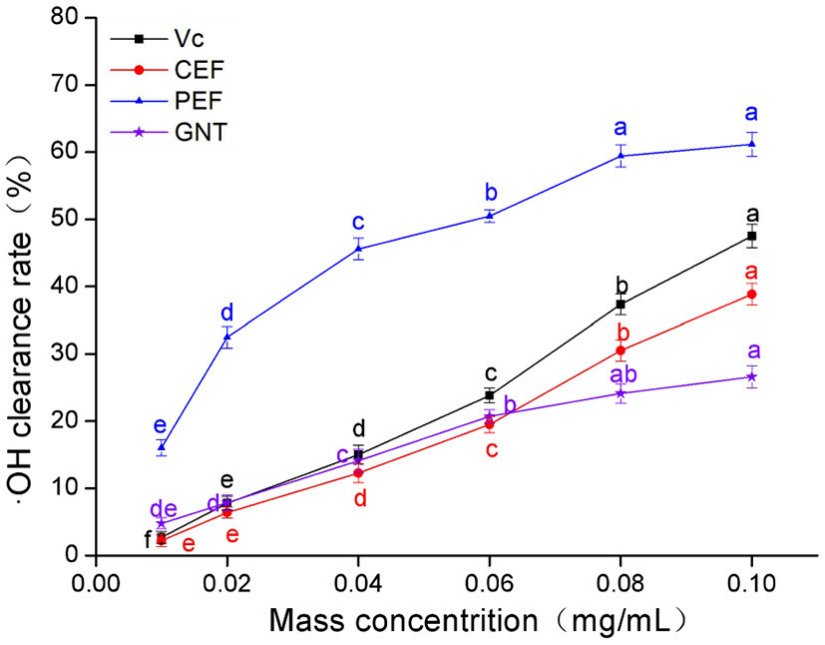
·OH clearance rate of CEF, PEF, and GNT at different concentrations. Different letters indicate significant differences in ·OH scavenging ability of the same substance at different concentrations (*p* < 0.05).

As shown in Figure 4, the clearance rate achieved by Vc, CEF, PEF, and GNT in each group was concentration-dependent. The trends were similar to those observed for DPPH·(Section DPPH· scavenging capacity analysis). The IC_50_ values were 0.061, 0.108, 0.137, and 0.219 mg/mL, respectively. The clearance rate in the CEF, PEF, and GNT groups increased significantly at concentrations of 0.01–0.06 mg/mL (*p* < 0.05), but increased slowly in the 0.06–0.10 mg/mL range. However, the scavenging capacity of PEF against ·OH was significantly greater than that of the other compounds. This activity was 1.57 times that of CEF, 1.28 times that of Vc, and 2.30 times that of GNT at a concentration of 0.10 mg/mL. Pan et al. (34) found that when the mass concentration of *Ipomoea aquatic Forsk* flavonoids was 0.10 mg/mL, the rate of ·OH clearance rate was less by 20%. The clearance rate of CEF and PEF was 35.15 and 61.15%, respectively, significantly higher than the experimental results. Hence, as a new leafy vegetable, *M. crystallinum* has a high antioxidant activity.

### O${}_{2}^{\text{–}}\cdot$ scavenging capacity analysis

**Figure 5:**
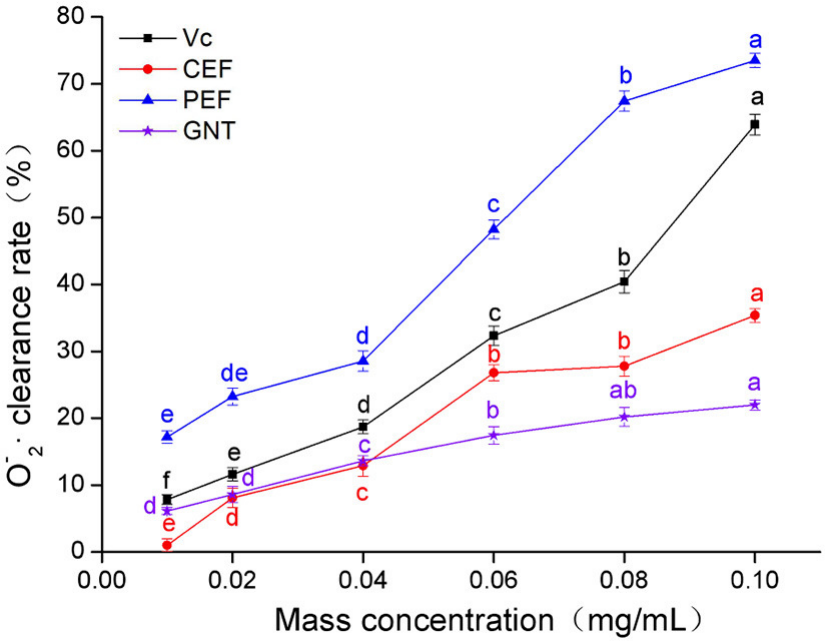
O${}_{2}^{-}\cdot$ clearance rate of CEF, PEF, and GNT at different concentrations. Different letters indicate significant differences in O${}_{2}^{-}\cdot$ scavenging ability of the same substance at different concentrations (*p* < 0.05).

As shown in Figure 5, when the concentration of the sample solutions ranged from 0.01 to 0.04 mg/mL, the O${}_{2}^{-}\cdot$ clearance rate was highest for PEF, followed by Vc, GNT, and CEF. In the PEF, Vc, and CEF groups, the clearance increased greatly at a concentration range of 0.04–0.10 mg/mL, while in the GNT group, this value increased slowly. The clearance rate of the GNT group was thus lower than that of the other groups at 0.04–0.10 mg/mL. The IC_50_ values of PEF, Vc, CEF, and GNT were 0.062, 0.088, 0.141, and 0.287 mg/mL, respectively. At a concentration of 0.10 mg/mL, the clearance rate of PEF against O${}_{2}^{-}\cdot$ was 2.08 times that of CEF, 1.15 times that of Vc, and 3.34 times that of GNT. Previous studies have shown that at a concentration of 0.10 mg/mL, the clearance rates of crude and refined flavonoids obtained from *Spinacia oleracea* against O${}_{2}^{-}\cdot$ are 17.03 and 22.66%, respectively (35). The clearance rates of CEF and PEF were significantly higher than the results above and were 35.35 and 73.48%, respectively. Together, the findings on the free radical scavenging ability of PEF against the three mentioned radicals indicated that PEF has high antioxidant activity *in vitro*. Therefore, we hypothesized that PEF could inhibit the oxidation and glycosylation of LDL.

### Comparative analysis of the CD production

**Figure 6:**
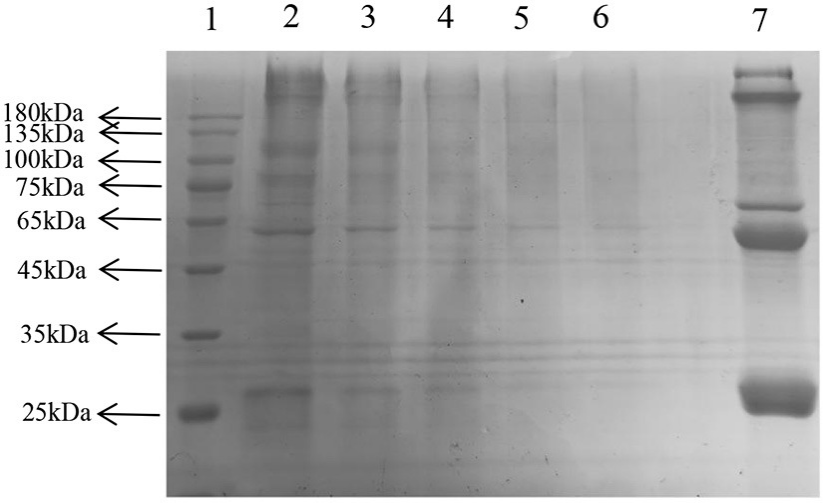
Sodium dodecyl sulfate—LDL polyacrylamide gel electrophoresis. Lane: 1, Prestained Protein Ladder; 2–6, LDL diluted to 1, 5, 10, 20, 30 times; 7, plasma.

SDS-PAGE showed several single bands between molecular weights of 10–180 kDa, indicating the some degree of purity of the extracted LDL (Figure 6) (25).

**Figure 7:**
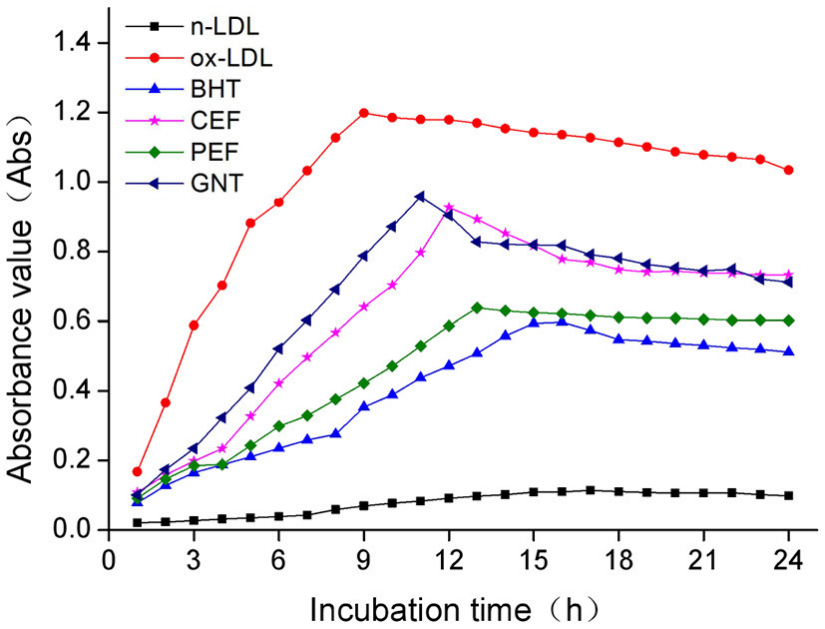
Kinetic curves of the CD generation and degradation in different oxidation incubation systems.

**Figure 8:**
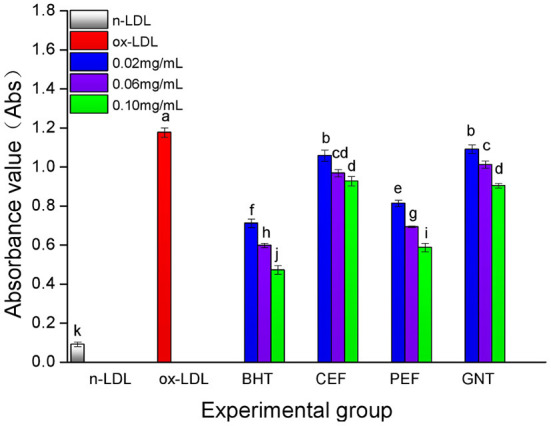
The CD generative effect of CEF, PEF and GNT at different concentrations. Different lowercase letters indicate significant differences between each individual experimental group (*p* < 0.05).

The process of LDL oxidation can be divided into three stages: delay, proliferation, and degradation (36). The dynamic change in the CD absorbance value reflects the early LDL oxidation inhibition effect of each incubation system. As shown in Figure 7, the absorbance values of the BHT, CEF, PEF, and GNT groups within 24 h were significantly lower than those of the ox-LDL group, indicating that these compounds presented different degrees of inhibition against CD production. The lag times of GNT, CEF, PEF, and BHT were 2, 3, 4, and 7 h, respectively, and the corresponding absorbance values were 0.958, 0.927, 0.638, and 0.598. The absorbance value in each group was negatively correlated with the mass concentration of the sample solution (*p* < 0.05) (Figure 8). Hence, together with the experimental results in Section DPPH· scavenging capacity analysis, the findings suggested that the flavonoids could remove the hydrogen peroxide radicals generated from LDL oxidation, thus inhibiting the lipid peroxidation caused by a free radical chain reaction and CD formation. Accordingly, they could inhibit the weak oxidation of LDL.

So far, no studies have investigated whether PEF can prevent AS by inhibiting LDL oxidation. Our results showed that PEF can effectively delay the CD generation time, reducing its production. These findings have an important theoretical significance for AS prevention.

### Comparative analysis of the TBARS production

**Figure 9:**
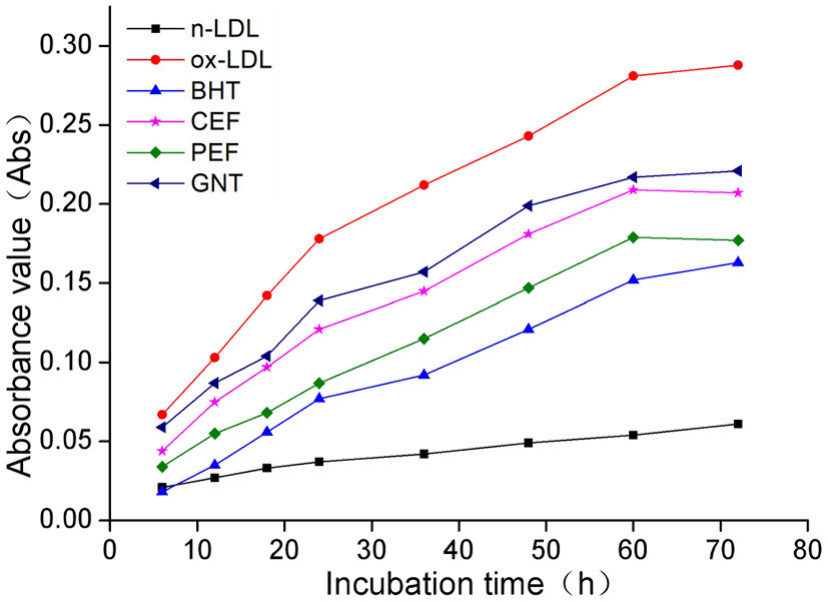
Kinetic curves of the TBARS generation and degradation in different oxidation incubation systems.

**Figure 10:**
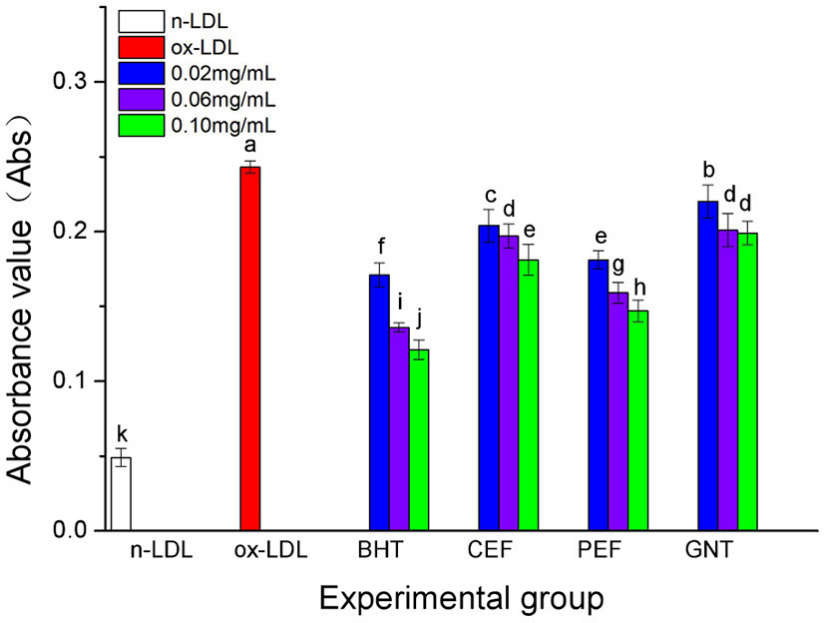
The TBARS generative effect of CEF, PEF, and GNT at different concentrations. Different lowercase letters indicate significant differences between each individual experimental group (*p* < 0.05).

When the lipid peroxidation reaction enters the degradation stage, a large number of peroxides such as lipid hydroperoxide (LOOH) and malondialdehyde (MDA) are produced. These covalently bind with total bile acid (TBA) to form TBARS, which shows specific absorption peaks at 535 nm. The read depth is proportional to the MDA concentration. Hence, the oxidative inhibition effect of different incubation systems on LDL can be evaluated by measuring the OD at specific wavelengths (37). The 72-h absorbance values in the BHT, CEF, PEF, and GNT groups were significantly lower than those in the ox-LDL group, indicating different degrees of inhibition against TBARS production (Figure 9). The absorbance of TBARS in each group increased gradually for the first 60 h. However, the rate of increase in the ox-LDL group was relatively high. This rate flattened and even decreased slightly after 60 h. At 72 h, the was highest for ox-LDL, followed by for GNT, CEF, PEF, BHT, and n-LDL. The corresponding absorbance values were 0.288, 0.221, 0.207, 0.177, 0.163, and 0.061, respectively. As for CD, the absorbance was inversely proportional to concentration, and the differences were significant (*p* < 0.05) (Figure 10).

When tangeretin, a single standard flavonoid was used, the inhibition rates against CD and TBARS were significantly lower than those of CEF and PEF, although the purity of the compound reached 95%. The flavonoids of *M. crystallinum* were extracted from the leaves of whole vegetables. Therefore, the crude extracts not only contained flavonoids but also polysaccharides and other small molecules, synergistically inhibiting the oxidation of LDL. Furthermore, the purified flavonoid components had a higher purity and relative abundance, thus exhibiting higher inhibitory activity. However, the content of nobiletin was found to be only second to that of tangeretin in PEF based on the UPLC-MS/MS results. Unlike general flavonoids, nobiletin was found to be lipid-soluble (38). Therefore, it could be speculated that PEF was more likely to enter the hydrophobic environment in which lipid peroxidation takes place, thus effectively inhibiting the oxidative modification of LDL. These results prove that the PEF unlike soybean isoflavones, which has no glycoside-binding flavonoids and can further inhibit LDL oxidation by suppressing TBARS production (11, 12).

### Comparative analysis of the LDL microstructure before and after oxidation

**Figure 11:**
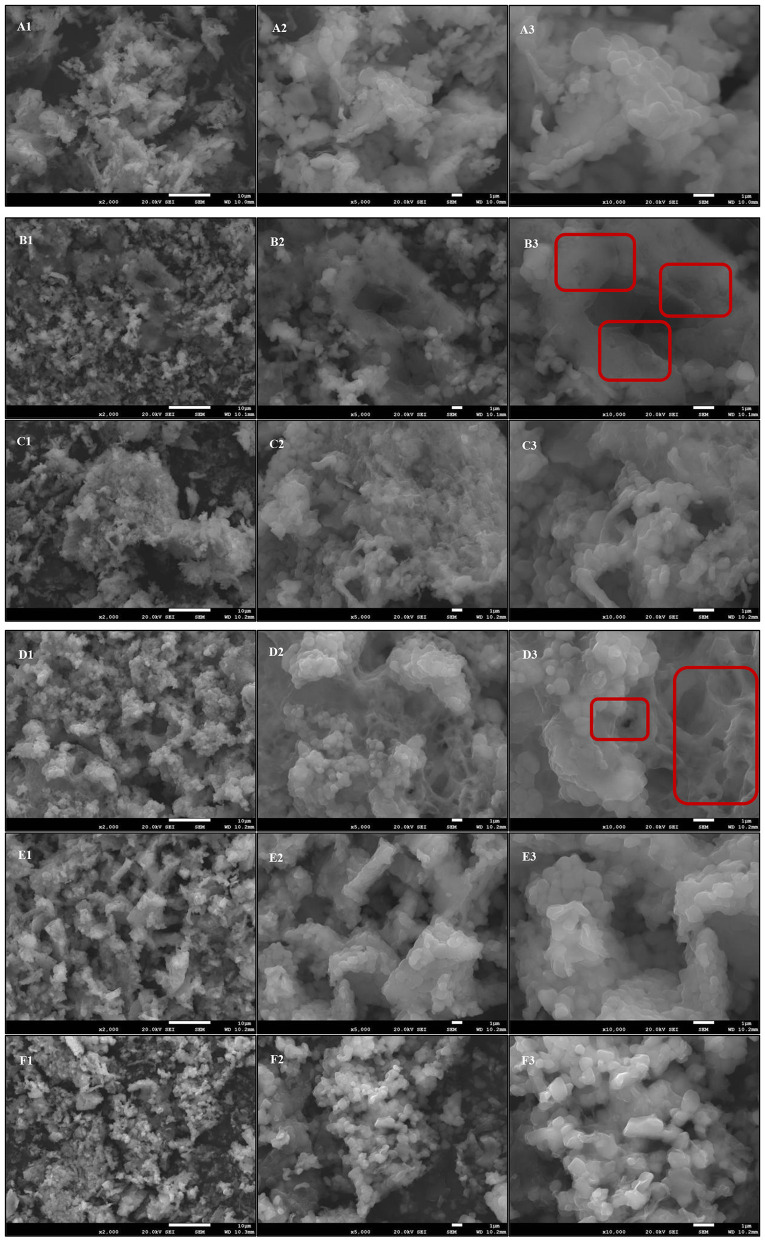
Comparison of the microstructure of different oxidation incubation systems. **(A)** n-LDL; **(B)** ox-LDL; **(C)** BHT; **(D)** CEF; **(E)** PEF; **(F)** GNT. Numbers: 1, × 2,000; 2, × 5,000; 3, × 10,000.

The microstructure of LDL treated in different oxidation incubation systems is shown in Figure 11. SEM results (×10,000) revealed that the surface structure in the n-LDL group was dense and smooth, while that in the ox-LDL group was partially damaged, with loosening and roughening and even deep holes observed on the surface (Figures 11A3,B3, respectively). The high degree of oxidation loss occurred because the oxidation products CD and TBARS gradually penetrated the inner core of LDL. The surface of LDL after the addition of BHT and PEF remained smooth and dense, with no obvious cavitation observed (Figures 11C3,E3, respectively). However, after the addition of GNT, the LDL surface appeared largely granular, and a large number of substances were found to be wrapped inside (Figure 11F3). This granular layer could rupture and cause serious surface damage if the reaction time is prolonged. The CEF group showed a larger degree of damage, and its surface was mostly reticular, with obvious cracks and pores. This led to the loosening and roughening of the granules (Figure 11D3). The red boxes in Figures 11B3,D3 show the oxidative damage in the ox-LDL and CEF group. These results indicated that PEF can effectively protect LDL from being eroded by oxidation products. In contrast, CEF has a weak protective effect because it contains more impurities, which could even accelerate the degree of damage, causing a large number of voids. GNT, as a single flavonoid standard, provided significantly weaker protection against LDL oxidation than PEF—the purified natural flavonoid extract obtained from the crude vegetable. To our knowledge, this experiment is the first time to demonstrate the inhibitory effect of flavonoids against oxidative damage to LDL at a microscopic level. The SEM findings further confirmed that PEF can inhibit LDL oxidation at a biochemical and structural level.

### Inhibition rate of Amadori products formation in the early stage

**Figure 12:**
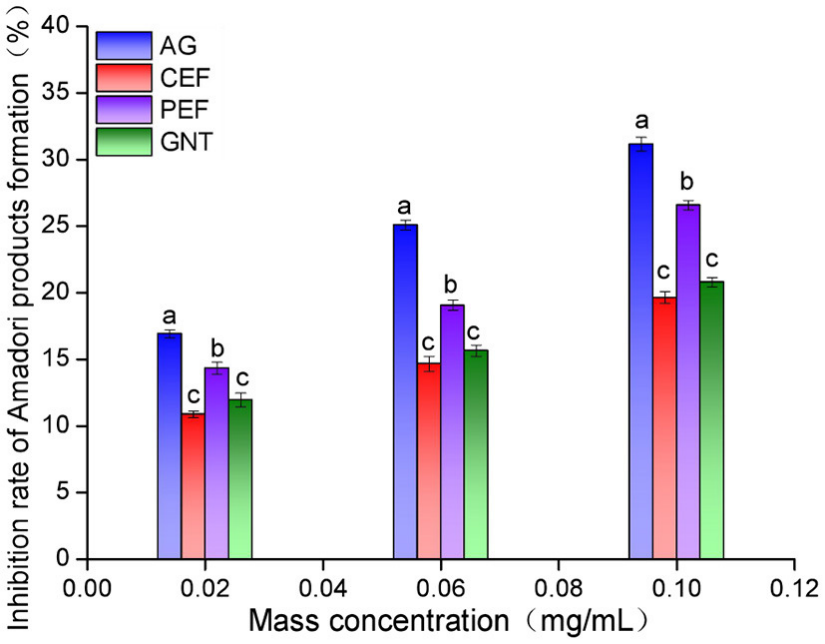
Amadori products early inhibition rate of CEF, PEF, and GNT at different concentrations. Different lowercase letters represents significant differences within each group at the same concentration (*p* < 0.05).

The non-enzymatic glycosylation of LDL can be divided into three stages. The early inhibition of glycosylation is reflected by a reduced amount of nitrotetrazolium blue chloride (NBT) (39). The inhibition rate was highest in the aminoguanidine bicarbonate (AG) group, followed by the PEF, GNT, and CEF group. Moreover, the inhibition was concentration-dependent (Figure 12). The corresponding inhibition rates at sample concentrations of 0.10 mg/mL were 31.15 ± 0.51%, 26.58 ± 0.35%, 20.78 ± 0.36%, and 19.64 ± 0.43%, respectively. The inhibitory rate of GNT was higher than that of CEF at different concentrations but significantly lower than that of PEF. This could be because PEF also contained other flavonoids besides tangeretin, such as nobiletin and farrerol, which have been reported to have strong antioxidant activity (40). Hence, the inhibition of Amadori product formation could arise from synergistic effects and lead to the prevention of LDL glycosylation.

### Inhibition rate of the dicarbonyl compound formation in the middle stage

**Figure 13:**
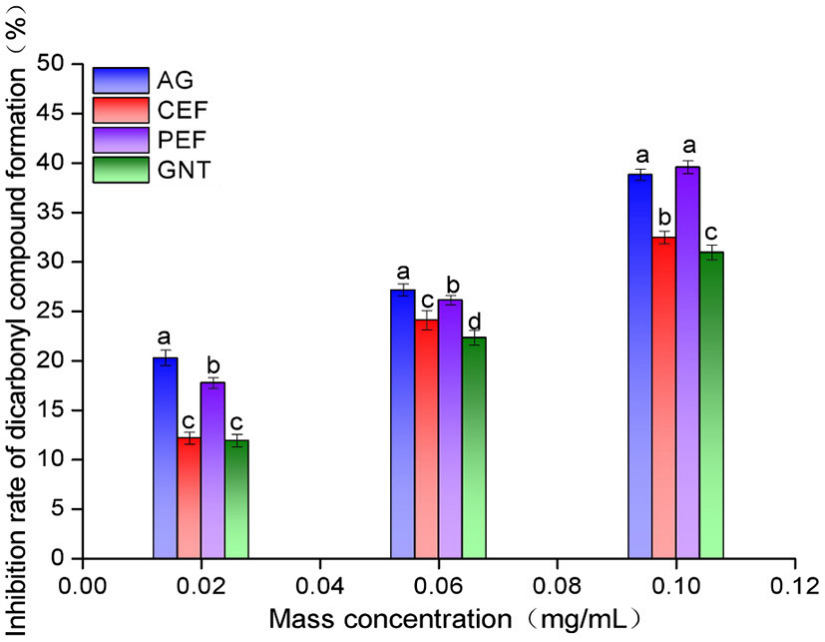
Intermediate dicarbonyl compounds inhibition rate of CEF, PEF, and GNT at different concentrations. Different lowercase letters represents significant differences within each group at the same concentration (*p* < 0.05).

The inhibition rates against dicarbonyl compound formation were highest for the AG glycosylation incubation system. This was followed by the incubation systems with PEF, CEF, and GNT. The rate of inhibition increased with sample concentration, and the significant differences between the AG and PEF groups gradually decreased (Figure 13). Even at a concentration of 0.10 mg/mL, the inhibition rate of PEF was slightly higher than that of AG (39.59 ± 0.65% vs. 38.83 ± 0.55%, respectively). Notably, the inhibition rate of each group against dicarbonyl compounds was significantly higher than that against Amadori products. This indicated that the inhibition of non-enzymatic glycosylation mainly occurred during the middle stages of the reaction. The significant differences between the inhibition achieved by equal concentrations of PEF, CEF, and GNT may be due to the differences in replacement positions and the quantities of aromatic compounds among the flavonoids. The role of flavonoids in the prevention of LDL glycosylation has not been reported before. We speculate that PEF could co-inhibit AS along with LDL oxidation. Our results showed that PEF can inhibit the formation of dicarbonyl compounds during glycosylation, which has important theoretical significance for the prevention of AS.

### The generation of the late AGEs

**Figure 14:**
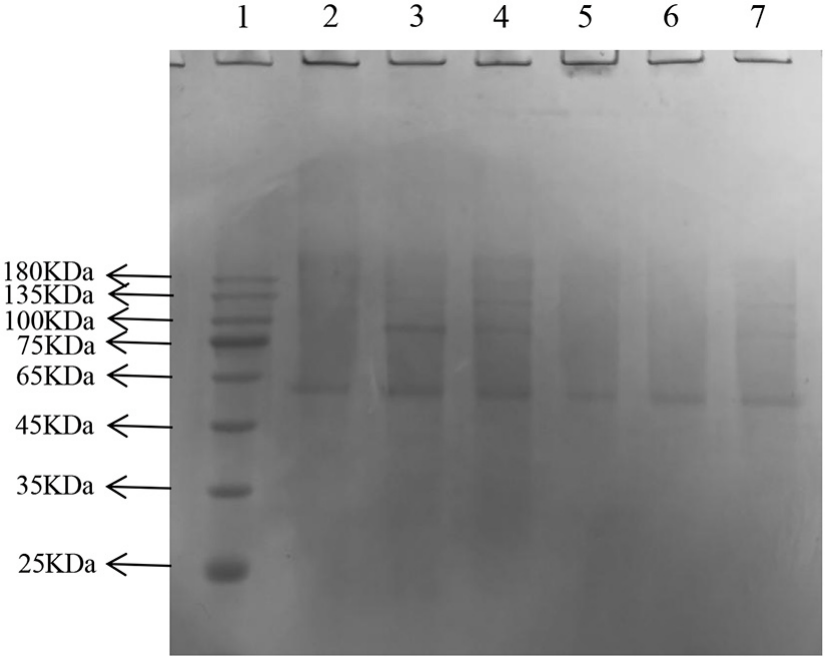
Migration of electrophoresis of AGEs in different incubation systems of glycation systems. Lane: 1, Prestained Protein Ladder; 2, n-gly-LDL; 3,GNT ; 4, gly-LDL; 5, CEF; 6, PEF; 7, AG.

In the later stage of glycosylation, the dicarbonyl compounds continue to generate irreversible end products called AGEs, which eventually cause AS. Moreover, AGEs are also the main contributor to diabetes and other associated complications (41). The depth of the electrophoretic bands directly reflected the amount of AGEs production, and the lighter the band, the lower the amount of production, and the more effective inhibition of glycosylation. After gel imaging system analysis, the bands of GNT were the darkest, followed by AG, CEF, and PEF, which indicated that glycosylation caused a significant decrease in the amount of cross-linked high molecular mass proteins, and CEF and PEF had different inhibitory effects on the generation of AGEs. Moreover, the inhibitory effect of GNT was not obvious (Figure 14). The trend of late AGEs inhibition was consistent with that for the inhibition of dicarbonyl compounds; i.e., the order of inhibition was PEF > CEF > GNT. The results further indicated that the inhibition of the glycosylation reaction mainly occurs in the middle and late stages. Hence, PEF can further inhibit the glycosylation of LDL by inhibiting the production of AGEs. We suspected that these findings were related to the content of flavonoids such as tangeretin, nobiletin, and farrerol in PEF. The compounds are polymethoxylated flavones that show cationic chelation, inhibiting glycosylation end product precursors by trapping the active carbonyl compound pyruvaldehyde and thus reducing AGE production (42). However, Matheus et al. (43) showed that *Syzygium cumini* leaf extracts had no effect on the increase in AGEs levels and eletrophoretic mobility, which are observed in LDL MG-glycated. We speculated that water extraction does not provide a high content of flavonoids and leads to more impurities. In contrast, PEF is a highly purified mixture. In addition, we also studied the degree of inhibition provided by PEF against the formation of Amadori products and dicarbonyl compounds at different glycosylation stages to comprehensively understand the inhibitory effect of flavonoids on LDL glycosylation. To our knowledge, this is the first report showing that flavonoids can modify LDL glycosylation.

## Conclusions

Research on the natural vegetable *M. crystallinum* has focused on cultivation and sedum acid metabolism. To the best of the author's knowledge, the flavonoid components had not been previously studied. This study demonstrated that *M. crystallinum* is rich in flavonoid compounds. Moreover, these flavonoids have strong radical scavenging activities and could potently inhibit the formation of CD and TBARS in LDL oxidation, while also inhibiting non-enzymatic LDL glycosylation. Meanwhile, most of the flavonoids are glycoside-free. To our knowledge, this study is the first to demonstrate the potent inhibition of LDL oxidation and glycosylation by glycoside-free flavonoids. These results indicate that vegetables such as *M. crystallinum* could be a rich source of functional flavonoids, which can be developed as natural hypoglycemic and hypolipidemic drugs or developed into functional foods. At present, our research is only *in vitro*, and there is a gap *in vivo* mechanistic research. So, in the future area of this study, we recommend being able to focus on the inhibition of *in vivo* LDL oxidation and glycosylation by these flavonoids components through animal experiments. These findings could establish the practical significance of these flavonoids components in preventing AS development and demonstrate their value in promoting health among humans.

## Data availability statement

The original contributions presented in the study are included in the article/Supplementary materials, further inquiries can be directed to the corresponding authors.

## Author contributions

MS and YW: software and formal analysis. MS and XF: conceptualization. MS and JF: methodology and writing-original draft preparation. XC and XF: resources and funding acquisition. MS: investigation and data curation. XF: validation and visualization. JF: writing-review and editing. XC: supervision and project administration. All authors have read and agreed to the published version of the manuscript.

## Funding

This research was funded by the Beijing Science and Technology Plan Development and Brand Building of Functional Fresh-cut Vegetable Products (Z181100009318001), Modern Agricultural Industry Technology System Beijing Leaf Vegetable Innovation Team (BAIC07-2021), and Beijing Innovation Consortium of Agriculture Research System (BAIC01-2022).

## Conflict of interest

Author XF was employed by Beijing Unong High-Quality Farm Products Planning Limited Company. The remaining authors declare that the research was conducted in the absence of any commercial or financial relationships that could be construed as a potential conflict of interest.

## Publisher's note

All claims expressed in this article are solely those of the authors and do not necessarily represent those of their affiliated organizations, or those of the publisher, the editors and the reviewers. Any product that may be evaluated in this article, or claim that may be made by its manufacturer, is not guaranteed or endorsed by the publisher.
