# Supplementary figures and images for: Composition, anti-LDL oxidation, and non-enzymatic glycosylation inhibitory activities of the flavonoids from Mesembryanthemum crystallinum
Source: Front Nutr. 2022 Sep 14;9:963858. doi: 10.3389/fnut.2022.963858 (PMC9521712; doi:10.3389/fnut.2022.963858)

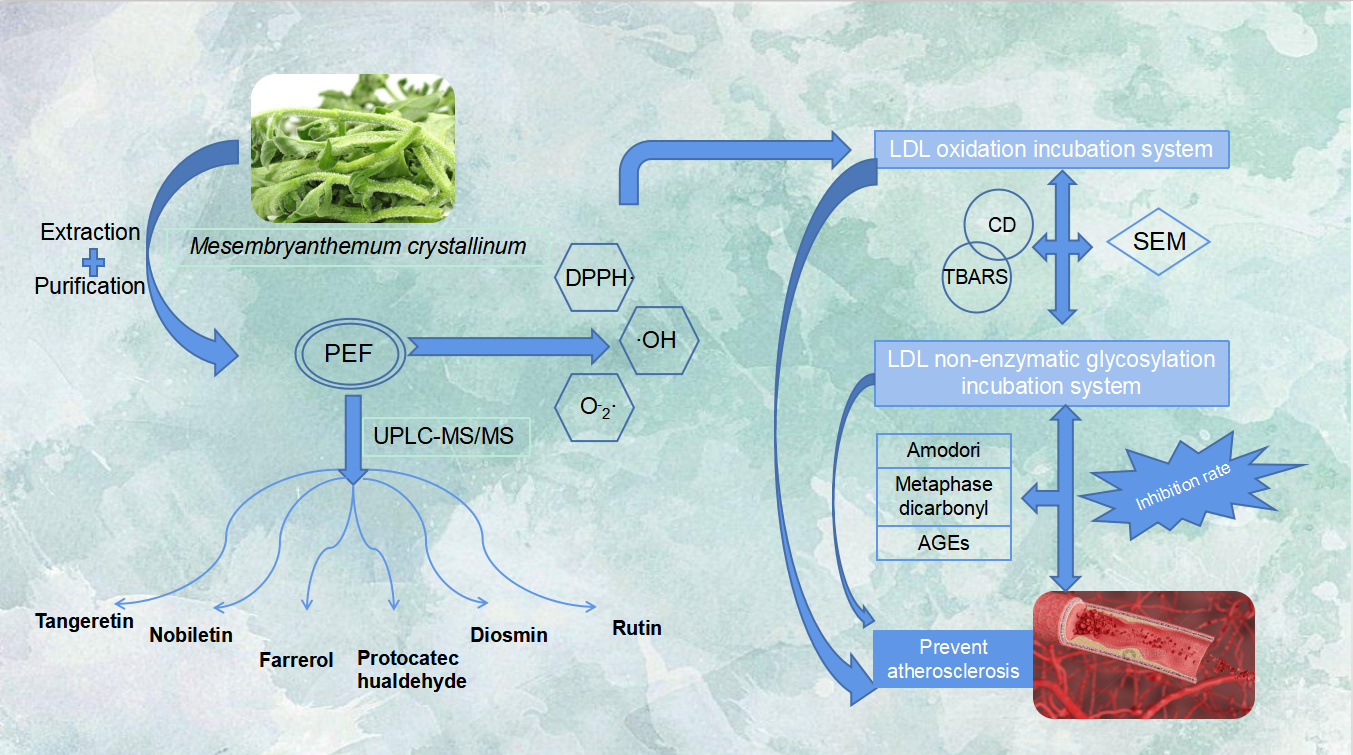

Supplement: Supplementary file 1 [file Image_1.PNG]
